# Five billion people unprotected from trans fat leading to heart disease

**Published:** 2023-03

**23 January 2023**  -  Five billion people globally remain unprotected from harmful trans fat, a new status report from WHO has found, increasing their risk of heart disease and death.

Since WHO first called for the global elimination of industrially produced trans fat in 2018 – with an elimination target set for 2023 – population coverage of best-practice policies has increased almost six-fold. Forty-three countries have now implemented best-practice policies for tackling trans fat in food, with 2.8 billion people protected globally.

Despite substantial progress, however, this still leaves 5 billion worldwide at risk from trans fat’s devastating health impacts with the global goal for its total elimination in 2023 remaining unattainable at this time.

Industrially produced trans fat (also called industrially produced trans-fatty acids) is commonly found in packaged foods, baked goods, cooking oils and spreads. Trans fat intake is responsible for up to 500 000 premature deaths from coronary heart disease each year around the world.

“Trans fat has no known benefit, and huge health risks that incur huge costs for health systems,” said WHO Director-General, Dr Tedros Adhanom Ghebreyesus. “By contrast, eliminating trans fat is cost effective and has enormous benefits for health. Put simply, trans fat is a toxic chemical that kills, and should have no place in food. It’s time to get rid of it once and for all.”

Currently, 9 of the 16 countries with the highest estimated proportion of coronary heart disease deaths caused by trans fat intake do not have a best-practice policy. They are Australia, Azerbaijan, Bhutan, Ecuador, Egypt, Iran (Islamic Republic of), Nepal, Pakistan and Republic of Korea.

Best-practices in trans fat elimination policies follow specific criteria established by WHO and limit industrially produced trans fat in all settings. There are two best-practice policy alternatives: 1) mandatory national limit of 2 grams of industrially produced trans fat per 100 grams of total fat in all foods; and 2) mandatory national ban on the production or use of partially hydrogenated oils (a major source of trans fat) as an ingredient in all foods.

“Progress in eliminating trans fat is at risk of stalling, and trans fat continues to kill people,” said Dr Tom Frieden, President and CEO of Resolve to Save Lives. “Every government can stop these preventable deaths by passing a best-practice policy now. The days of trans fat killing people are numbered – but governments must act to end this preventable tragedy.”

While most trans fat elimination policies to date have been implemented in higher-income countries (largely in the Americas and in Europe), an increasing number of middle-income countries are implementing or adopting these policies, including Argentina, Bangladesh, India, Paraguay, Philippines and Ukraine. Best-practice policies are also being considered in Mexico, Nigeria and Sri Lanka in 2023. If passed, Nigeria would be the second and most populous country in Africa to put a best-practice trans fat elimination policy in place. No low-income countries have yet adopted a best-practice policy to eliminate trans fat.

In 2023, WHO recommends that countries focus on these four areas: adopting best-practice policy, monitoring and surveillance, healthy oil replacements and advocacy. WHO guidance has been developed to help countries make rapid advances in these areas.

WHO also encourages food manufacturers to eliminate industrially produced trans fat from their products, aligning to the commitment made by the International Food and Beverage Alliance (IFBA). Major suppliers of oils and fats are asked to remove industrially produced trans fat from the products sold to food manufacturers globally.

The report, called Countdown to 2023 WHO Report on global trans fat elimination 2022, is an annual status report published by WHO in collaboration with Resolve to Save Lives, to track progress towards the goal of trans fat elimination in 2023.

Available from: https://www.who.int/news/item/23-01-2023-five-billion-people-unprotected-from-trans-fat-leading-to-heart-disease

